# Reverse Transcriptase Connection and RNase H Domain Variation in HIV-1 Subtype C Among Individuals with Virologic Failure in Botswana

**DOI:** 10.3390/biomedicines14091915

**Published:** 2026-08-26

**Authors:** Boitumelo Janet L. Zuze, Wonderful T. Choga, Natasha O. Moraka-Mankge, Segomotso Maphorisa, Modiegi Mothudi, Maruping Maruping, Thato Phuthego, Margaret Mokomane, Sikhulile Moyo, Simani Gaseitsiwe

**Affiliations:** 1 Botswana Harvard Health Partnership, Gaborone Private Bag BO 320, Botswana; bzuze@bhp.org.bw (B.J.L.Z.); wchoga@bhp.org.bw (W.T.C.); nmoraka@bhp.org.bw (N.O.M.-M.); smaphorisa@bhp.org.bw (S.M.); msebika@bhp.org.bw (M.M.); mjkmaruping@gmail.com (M.M.); smoyo@bhp.org.bw (S.M.); 2 Department of Medical Laboratory Sciences, School of Allied Health Sciences, University of Botswana, Gaborone Private Bag UB 0022, Botswana; phuthegot@ub.ac.bw (T.P.); mokomanem@ub.ac.bw (M.M.); 3 National Health Laboratory, Ministry of Health, Gaborone Private Bag 0057, Botswana; 4 Department of Immunology and Infectious Diseases, Harvard T. H. Chan School of Public Health, Harvard University, Boston, MA 02115, USA; 5 Division of Medical Virology, Department of Pathology, Stellenbosch University, Cape Town P.O. Box 241, South Africa; 6 School of Health Systems and Public Health, University of Pretoria, Private Bag X20, Hatfield, Pretoria 0002, South Africa

**Keywords:** HIV-1C, reverse transcriptase connection, RNase H, drug resistance mutations (DRMs), virologic failure (VF), Oxford Nanopore sequencing, minority variants, molecular surveillance, Botswana

## Abstract

**Background:** Classical reverse transcriptase (RT) drug resistance mutations (DRMs) are primary determinants of antiretroviral treatment failure, but polymorphisms within the RT connection and RNase H domains may also influence RT inhibitor susceptibility. These regions remain poorly characterised in HIV-1 subtype C (HIV-1C), the predominant subtype in sub-Saharan Africa. This study investigated RT connection and RNase H polymorphisms in treatment-experienced people with HIV (PWH) experiencing virologic failure (VF) in Botswana. **Methods:** Seventeen plasma samples from the Botswana National HIV Drug Resistance Programme with documented VF (viral load >200 copies/mL) and prior Sanger resistance results were selected. One sample was excluded because of insufficient Oxford Nanopore Technologies (ONT) sequencing coverage, leaving 16 samples for analysis. Samples were amplified using the DeepChek^®^ HIV-1 Full PR/RT/INT assay and analysed using the HIVgenomeR™ v2.0 ONT pol DRM pipeline. ONT-derived RT resistance profiles were compared with historical Sanger results, and RT connection and RNase H polymorphisms were characterised. **Results:** ONT identified additional RT DRMs in five sequences previously classified as lacking classical RT DRMs. Of the remaining six sequences without classical DRMs, four harboured minority RT variants. RT connection polymorphisms were common, including G335D (13/16) and T377M (9/16). Recurrent mutation combinations included T377M–T470A, A371V–E399D, T377M–E399D, and T377M–A360T. **Conclusions:** The extended HIV-1 pol genotyping assay enabled characterisation of the RT connection and RNase H domains while improving detection of classical RT DRMs and minority variants. These findings support further investigation of extended RT sequencing for HIV-1C molecular surveillance.

## 1. Introduction

Despite the widespread scale-up of antiretroviral therapy (ART), virologic failure (VF) remains a challenge in human immunodeficiency virus (HIV) treatment programmes, particularly in sub-Saharan Africa (SSA), where HIV-1 subtype C (HIV-1C) predominates [1,2,3,4,5]. Standard clinical management of VF relies on genotypic resistance testing targeting key viral enzymes, including protease (PR), integrase (IN) and reverse transcriptase (RT). These assays are used to detect classical drug resistance mutations (DRMs) associated with resistance to protease inhibitors (PIs), integrase strand transfer inhibitors (INSTIs) and nucleoside and non-nucleoside reverse transcriptase inhibitors (NRTIs and NNRTIs), respectively [6]. These mutations are well characterised and form the basis for most treatment-switch decisions.

Of all the targets, RT remains the most extensively studied due to its central role in viral replication and the long-standing use of NRTI and NNRTI in first- and second-line ART regimens. However, resistance testing is focused on the RT domain, while other functionally important regions of RT, particularly the connection and RNase H domains, are not routinely included in standard genotyping assays [7,8,9,10].

A subset of people with HIV (PWH) experiencing VF appear to lack detectable classical RT resistance mutations following routine genotypic resistance testing. While advances in next generation sequencing (NGS) have demonstrated that some apparently wildtype viruses harbour low-frequency or previously undetected resistance mutations, VF may still occur in cases where classical RT DRMs remain absent [11,12]. Although suboptimal adherence, pharmacokinetic variability and archived resistance mutations not detected in plasma may also contribute to VF in these individuals, additional viral genetic factors beyond canonical resistance-associated sites are increasingly being investigated [13,14]. In particular, substitutions occurring outside standard resistance testing regions, within the RT connection and RNase H domains, remain poorly understood despite growing evidence that they may influence reverse transcription efficiency, enzyme processivity and viral replication capacity [10,13].

The RT connection (~aa 321–440) links the polymerase and RNase H domains (~aa 441–560) of RT and contributes to structural stability and functional coordination of the enzyme. The RNase H domain is essential for degradation of the RNA template during reverse transcription, enabling synthesis of double-stranded viral DNA. Substitutions within these regions have been reported to influence the effects of classical resistance mutations, compensate for fitness costs and also potentially contribute to reduced susceptibility to antiretroviral drugs [8,13,15]. However, most available evidence is from HIV-1B, while the role of these regions in HIV-1C, which is the most prevalent subtype in southern Africa and globally, remains comparatively undercharacterised [16,17,18]. Furthermore, several RT connection substitutions, including G335D, are recognised as naturally occurring residues in HIV-1C, highlighting the importance of distinguishing subtype-specific background variation from mutations potentially associated with treatment failure [19].

The contribution of RT connection and RNase H polymorphisms to VF in PWH without classical resistance mutations therefore remains unclear. It is not known whether these substitutions occur as isolated events with limited functional consequences or form co-occurring mutational patterns that may reflect coordinated viral adaptation under drug pressure [13,15,20].

To address these gaps, this study investigated the landscape of the RT connection and RNase H domains in HIV-1C sequences from PWH experiencing VF in Botswana. These analyses primarily aimed to evaluate differences between PWH with and without classical RT drug resistance mutations and to identify recurrent and co-occurring polymorphisms within these regions. The study was motivated by the observation that VF can occur in a subset of PWH in the absence of classical RT resistance mutations, suggesting that additional viral genetic factors may contribute to treatment failure. Understanding these patterns may provide further insight into alternative mechanisms underlying VF in HIV-1C.

## 2. Materials and Methods

### 2.1. Study Design and Sample Selection

This retrospective cross-sectional study investigated RT connection and RNase H domain polymorphisms in HIV-1C from PWH experiencing VF within the Botswana National HIV Treatment Programme. Plasma samples collected between 2019 and 2025 and stored at −80 °C were retrieved from the Botswana National HIV Drug Resistance Repository. Samples were selected from treatment-experienced (TE) individuals with documented ART regimen histories, confirmed VF and available historical Sanger-based HIV drug resistance test results.

Seventeen plasma samples meeting the inclusion criteria were selected for re-sequencing. VF was defined according to the Botswana National HIV Treatment Guidelines as a two consecutive plasma HIV-1 viral load (VL) > 200 copies/mL after at least 6 months on ART [21,22]. Historical Sanger resistance reports were reviewed to determine the presence or absence of classical RT DRMs, identifying five DRM-positive (DRM+) and twelve lacking classical RT DRMs (DRM−) sequences.

**Scheme 1:**
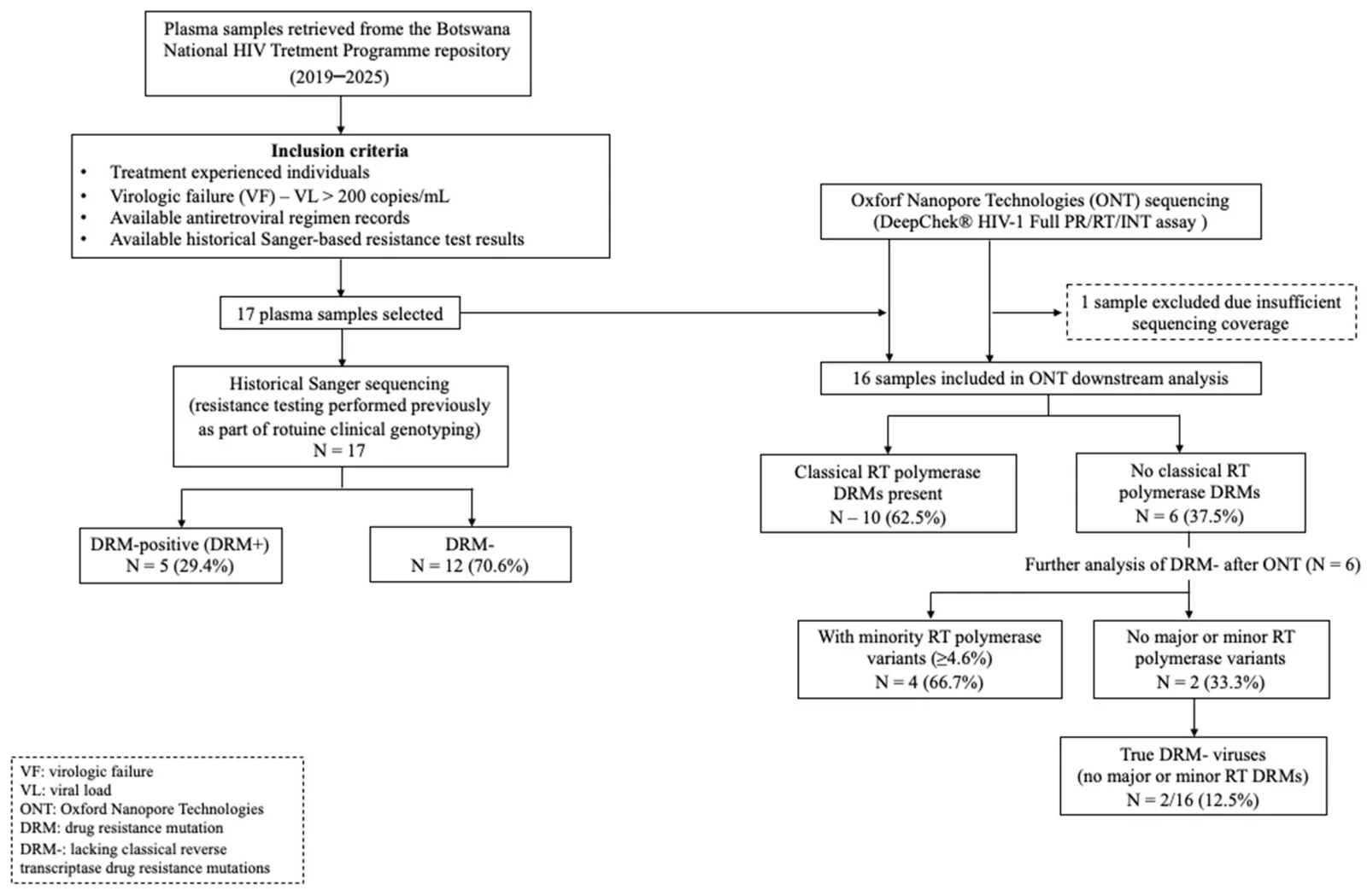
This is a schema used for sample selection, sequencing and classification of HIV-1C virologic failure cases.

The 17 samples were subsequently sequenced using Oxford Nanopore Technologies (ONT). One sample failed because of insufficient sequence coverage and was excluded from the downstream analyses. Consequently, 16 sequences were included in the final analyses. The sample selection workflow is summarised in Scheme 1.

### 2.2. Viral RNA Extraction, Amplification and Sequencing

Viral RNA was extracted from plasma using the QIAamp Viral RNA Mini Kit (Qiagen, Hilden, Germany) according to the manufacturer’s instructions. Amplification of the HIV-1 pol region encompassing the PR, RT, and IN genes was performed using the DeepChek^®^ Assay HIV-1 Full PR/RT/INT Drug Resistance V2.x (ABL Diagnostics, Luxembourg) according to the manufacturer’s protocol [23,24].

Sequencing libraries were prepared using the ONT Rapid Barcoding Kit (Oxford Nanopore Technologies plc, Oxford, UK) according to the manufacturer’s instructions. Sequencing was performed on an ONT GridIon platform using a Flongle Flow Cell (R10.4.1) to generate high-coverage HIV-1 pol sequences for resistance and polymorphism analyses.

### 2.3. Sequence Analysis

Raw sequencing data were analysed using the in-house HIVgenomeR™ v2.0 ONT pol DRM pipeline. Sequence alignment was performed using a dual-mapping strategy against the best-matching HIV-1 subtype reference and HXB2 reference genome, with mutation annotation reported using HXB2 codon numbering.

Consensus sequences were generated and screened for DRMs within the RT region. Major DRMs were defined according to established HIV drug resistance interpretation algorithms implemented within the pipeline. Variant calls were classified according to predefined frequency thresholds. Minority variants were defined as amino acid substitutions detected at frequencies >4.5% and <20% of sequencing reads, in accordance with the reporting thresholds implemented in the HIVgenomeR™ v2.0 ONT pol DRM pipeline. The pipeline incorporates the Stanford HIV Drug Resistance Database (v9.4) together with curated RT connection and RNase H residues from the literature [13,25,26,27]. Variants detected at frequencies ≥20% were considered consensus-level variants, whereas those detected at frequencies 4.5% and below were classified as trace variants. Only variants meeting the pipeline quality criteria (≥20 mapping quality, ≥7 base quality, ≥10× sequencing depth and ≥3 alternative reads) were retained for downstream analyses.

Sequences were evaluated for amino acid substitutions within the RT connection and RNase H domains. Mutation interpretation accounted for HIV-1C-specific amino acid variation while retaining HXB2 codon numbering for mutation annotation. Particular attention was given to substitutions previously reported to influence RT inhibitor (RTI) susceptibility or to compensate for fitness costs associated with classical RT resistance mutations [7,13,26,27,28].

### 2.4. Classification of Sequences

Samples were initially classified according to previous Sanger sequencing results as either DRM-positive or DRM−. Following ONT sequencing, all sequences were reclassified according to the presence or absence of classical RT DRMs, and all sequences were assessed for minority variants as well as RT connection and RNase H polymorphisms.

### 2.5. Mutation Frequency and Co-Occurrence Analysis

The prevalence of RT connection and RNase H polymorphisms was calculated across the study population. Recurrent substitutions were identified based on their occurrence in multiple individuals. Co-occurrence analysis was performed using consensus-level RT connection and RNase H polymorphisms (frequency ≥ 20%). Co-occurring mutation patterns were evaluated by assessing substitutions that repeatedly occurred together within the same viral sequence. Particular attention was given to accessory mutation clusters previously associated with reduced susceptibility to RTIs.

### 2.6. Statistical Analysis

Descriptive statistics were used to summarise mutation frequencies and sequence classifications. Categorical variables were reported as counts and percentages. The prevalence of RT connection and RNase H polymorphisms was calculated for the overall cohort and among DRM-positive and DRM− groups. Jaccard similarity analysis was used as an exploratory descriptive approach to identify recurrent mutation co-occurrence patterns. No formal statistical significance testing or correction for multiple comparisons was performed.

## 3. Results

### 3.1. Cohort and Sequencing Outcomes

Sixteen plasma samples from PWH experiencing VF were included in the study following ONT sequencing and quality control. Historical Sanger sequencing had classified five sequences as harbouring classical RT DRM (DRM+) and eleven as DRM−. Sample selection and classification are summarised in Scheme 1. Re-analysis using ONT confirmed all five previously identified DRM+ sequences and detected additional NRTI and NNRTI DRMs in five sequences previously classified as DRM− (Sequences 3, 9, 12, 14 and 16). Consequently, the number of DRM+ sequences increased from five to ten following ONT sequencing (Table 1). Among the six sequences that remained DRM− after ONT analysis, four harboured minority RT variants detected at frequencies ≥ 4.6% (Sequences 1, 5, 13 and 15). Only two sequences (Sequences 4 and 10) lacked both major and minority RT DRMs. Comprehensive resistance profiles, including historical Sanger classification, PR, IN, RT mutations, RT connection and RNase H polymorphisms, and current ART regimens are presented in Table 1 and Table S1.

### 3.2. Prevalence of RT Connection and RNase H Polymorphisms

**Figure 1:**
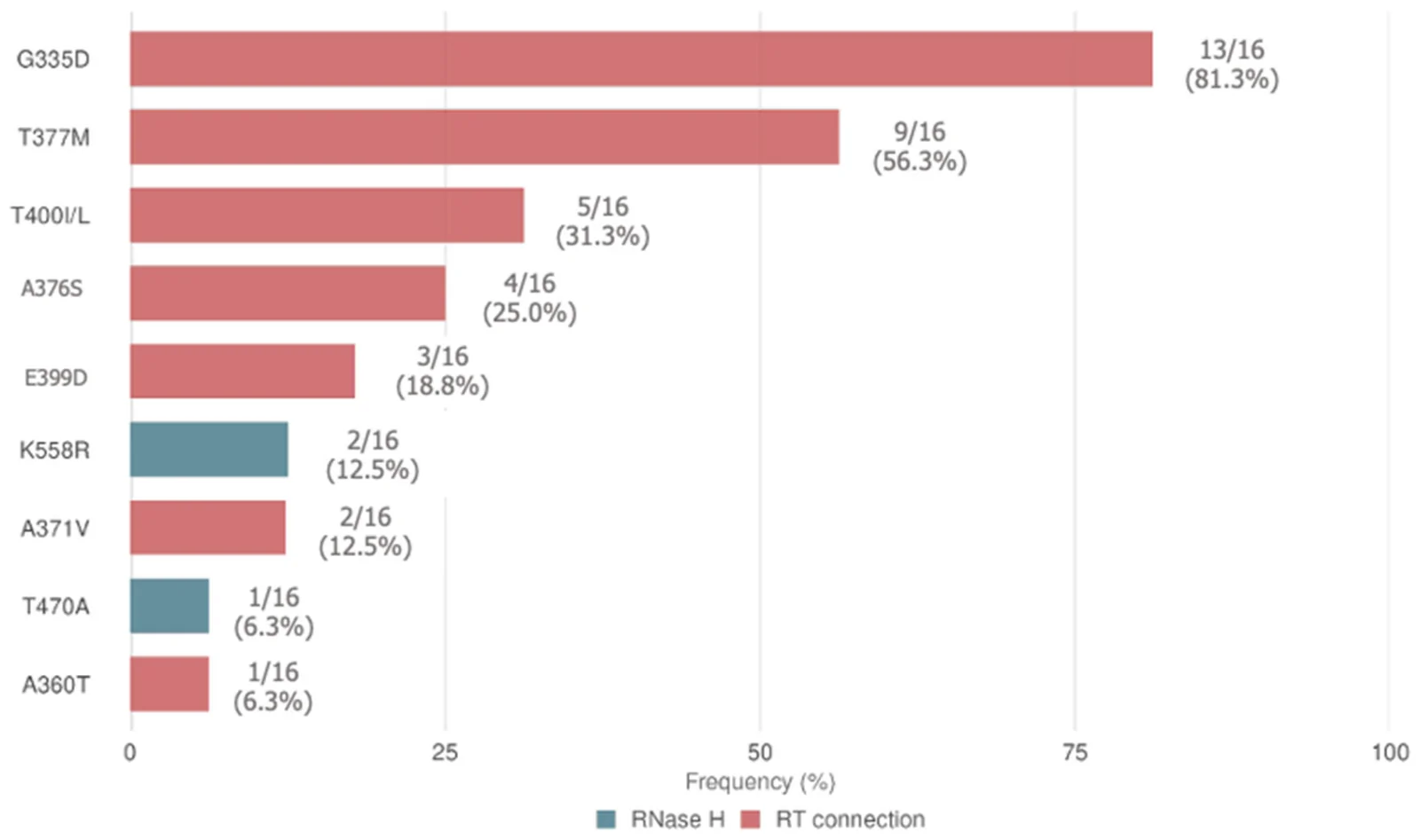
Prevalence of RT connection and RNase H polymorphisms identified in HIV-1 subtype C sequences from people with HIV experiencing virologic failure in Botswana: Frequency of amino acid substitutions identified within the RT connection and RNase H domains among 16 HIV-1C sequences from PWH experiencing VF in Botswana.

RT connection domain polymorphisms were common across the study cohort. The most prevalent substitution was G335D, detected in 13 of 16 sequences, followed by T377M, identified in 9 of 16 sequences. Other recurrent RT connection substitutions included A376S (4/16), E399D (3/16), T400I/L (5/16), A371V (2/16) and A360T (1/16). Within the RNase H domain, K558R was observed in 2/16 sequences, including one minority variant present at 24.0%, while T470A occurred in 1/16 sequences. Overall, polymorphisms were concentrated within the RT connection domain, whereas RNase H substitutions occurred less frequently (Figure 1). Importantly, RT connection polymorphisms were detected across all resistance categories, including sequences harbouring classical RT DRMs, sequences containing only minority variants and sequences lacking detectable RT resistance mutations altogether.

### 3.3. Co-Occurrence Structure of RT Connection and RNase H Polymorphisms

**Figure 2:**
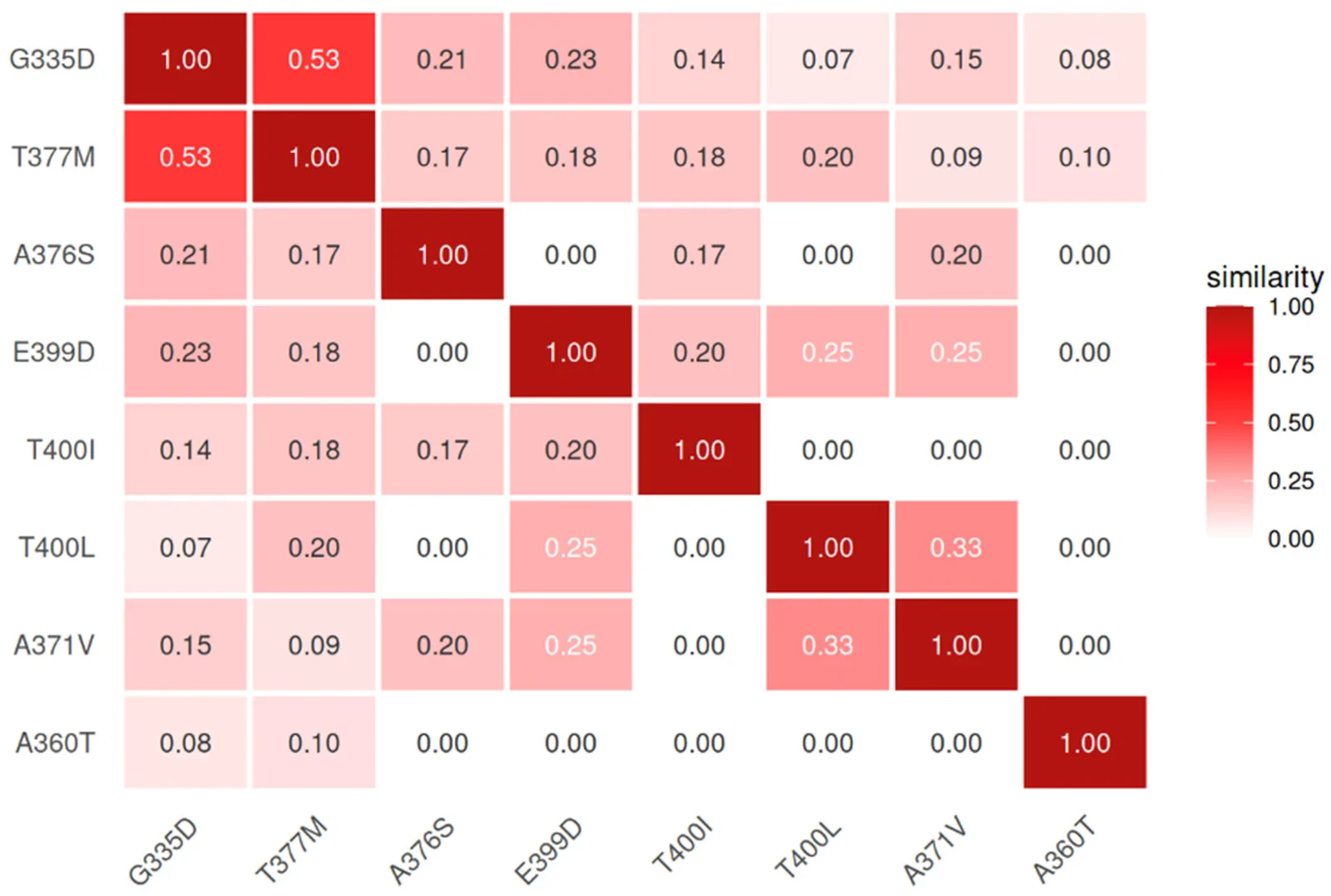
Jaccard similarity heatmap of RT connection and RNase H polymorphisms: Heatmap showing pairwise Jaccard similarity coefficients between amino acid substitutions identified within the RT connection and RNase H domains. Colour intensity reflects increasing co-occurrence strength.

Jaccard similarity analysis demonstrated structured co-occurrence patterns among RT connection and RNase H polymorphisms (Figure 2). The strongest pairwise similarity was observed between G335D and T377M (Jaccard index = 0.53). Moderate co-occurrence was also observed between A371V and T400L (0.33), E399D and A371V (0.25), E399D and T400L (0.25), and G335D and E399D (0.23). Lower similarity values were observed for the remaining mutation pairs, indicating that only a subset of polymorphisms consistently co-occurred across the cohort (Figure 2). Overall, these findings demonstrate that RT connection polymorphisms were organised into recurrent co-occurring patterns rather than occurring independently.

### 3.4. Accessory RT Connection and RNase H Mutation Clusters

**Figure 3:**
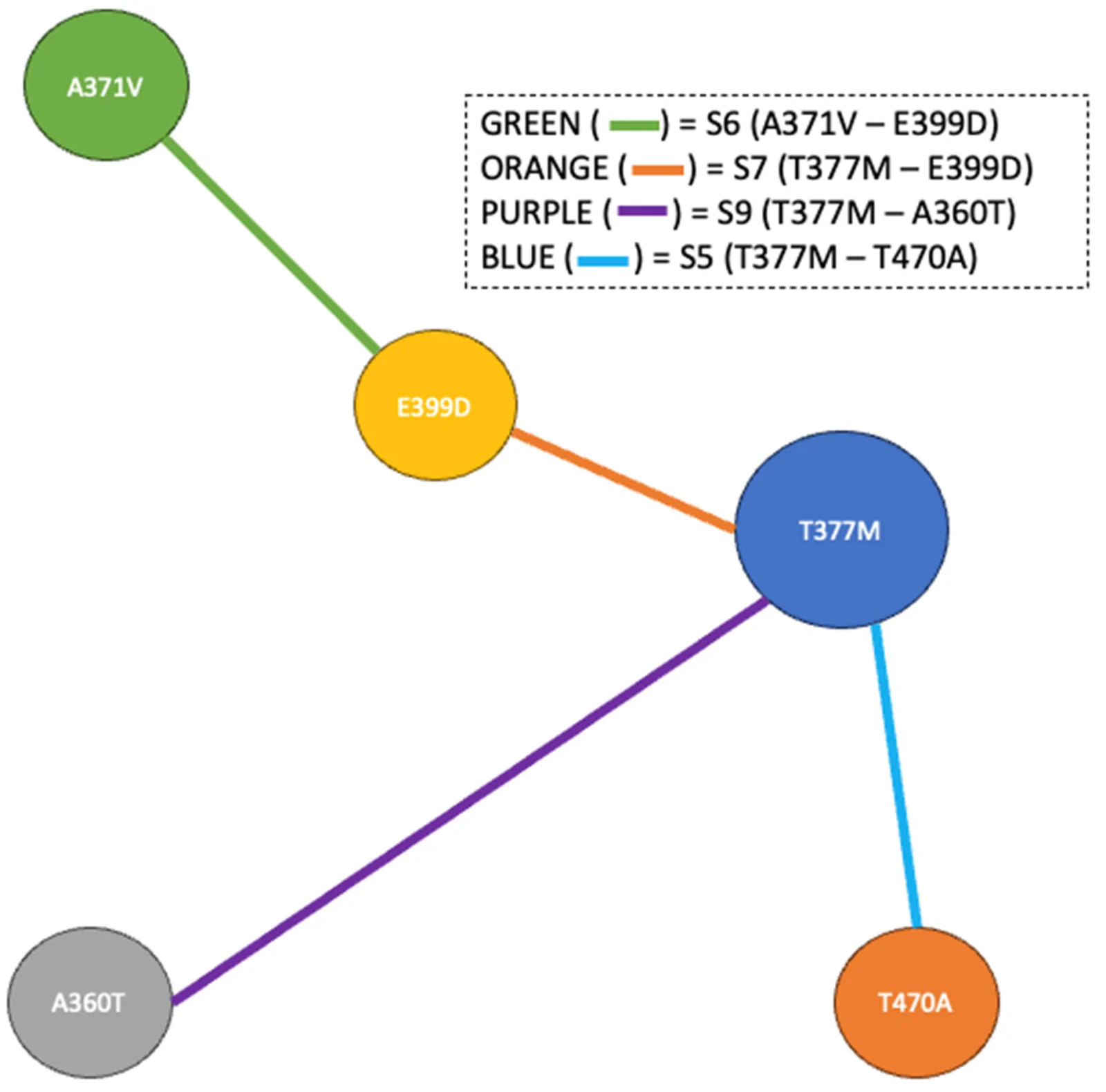
Recurrent accessory mutation clusters identified within the RT connection and RNase H domains: Nodes represent amino acid substitutions and coloured edges represent accessory mutation pairs identified in individual HIV-1C sequences. Green = S6 (A371V–E399D); orange = S7 (T377M–E399D); purple = S9 (T377M–A360T); blue = S5 (T377M–T470A). These mutation pairs have previously been associated with reduced susceptibility to RTIs when present together.

In addition to the observed polymorphism and co-occurrence patterns, the HIVgenomeR™ v2.0 ONT pol DRM pipeline identified four accessory mutation clusters within the RT connection and RNase H domains that have previously been associated with reduced susceptibility to RTIs when present together (Figure 3). These included A371V–E399D (Sequence 6), T377M–E399D (Sequence 7), T377M–A360T (Sequence 9) and T377M–T470A (Sequence 5). Three of the four identified accessory clusters contained T377M, highlighting its recurrent occurrence within functionally annotated mutation combinations (Figure 3). Notably, these accessory clusters were detected in both DRM-positive and DRM− sequences, indicating that potentially relevant RT connection and RNase H mutation combinations are not restricted to sequences harbouring classical RT resistance mutations.

## 4. Discussion

This study demonstrates that the RT connection and RNase H domains harbour recurrent polymorphisms and accessory mutation clusters in HIV-1C from PWH experiencing VF in Botswana. Using ONT sequencing, we showed that extended RT analysis not only improved detection of classical RT DRMs, but also revealed structured patterns of RT connection and RNase H variation that persisted in sequences lacking detectable RT DRMs. These findings suggest the hypothesis that extended RT domains may contribute to the molecular complexity underlying VF in HIV-1C and warrant further investigation in larger cohorts and functional studies [6].

A key finding was the reclassification of resistance profiles following ONT sequencing. Historical Sanger sequencing identified classical RT DRMs in only five sequences, whereas ONT detected additional major DRMs in five previously classified DRM sequences. Furthermore, minority RT variants were identified in four of the remaining six DRM sequences, demonstrating the improved sensitivity of deep sequencing for detecting low-frequency resistant variants that may contribute to treatment failure, although their clinical significance requires further investigation [11,12].

Despite improved detection of classical RT mutations, RT connection polymorphisms remained highly prevalent across the cohort. G335D and T377M were the dominant substitutions, identified in 13/16 and 9/16 of sequences, respectively. These substitutions occurred not only alongside classical RT DRMs but also in sequences lacking detectable RT resistance, suggesting that the RT connection domain may represent an additional source of genetic variation during VF, although its direct contribution to treatment outcomes has yet to be established [8,10,13].

The present study builds on our previous characterisation of RT connection and RNase H polymorphisms in HIV-1C from Botswana [20]. While the previous study provided a broader overview of polymorphism diversity across pre-ART, treatment-naïve post-ART and TE populations, the present study focused specifically on TE individuals with VF. Despite differences in the polymorphism profiles observed, G335D was identified in both studies, consistent with its high background frequency in HIV-1C and supporting its interpretation as a recurrent HIV-1C polymorphism in Botswana rather than a virologic failure-specific mutation.

The recurrence of G335D in independent Botswana datasets is noteworthy because, although G335D has previously been reported as a resistance-associated mutation within the RT connection domain in HIV-1B, aspartic acid (D) represents the consensus amino acid at position 335 in HIV-1C rather than a mutation acquired during therapy [10,16,18]. According to the Stanford HIV Drug Resistance Database, while glycine (G) is the overall wild-type residue, HIV-1C predominantly harbours D at this position (85%), with glutamic acid (E) and serine (S) occurring at much lower frequencies (2.2% and 1.3%, respectively) [19]. Therefore, G335D may be considered a naturally occurring HIV-1C polymorphism rather than an acquired resistance mutation in this population. Similarly, position 377 show considerable natural variability within HIV-1C, with methionine (M) and leucine (L) representing the most common residues (42% and 39%, respectively), followed by glutamine (Q), isoleucine (I), valine (V) and arginine (R) [19]. While all participants in the present study were TE, the high prevalence of G335D and T377M is likely to reflect the underlying HIV-1C genetic composition rather than ART selection alone. Whether these naturally occurring polymorphisms modify RT inhibitor susceptibility or influences viral fitness independently or in combination with other RT mutations remains uncertain.

To date, relatively few studies have characterised RT connection and RNase H polymorphisms in HIV-1C from SSA. Among the available studies, Brehm et al. (2012) reported the frequent emergence of the RT connection mutation N348I among South African individuals experiencing first-line treatment failure, where it was associated with reduced susceptibility to multiple RT inhibitors [29]. In the present study, N348I was not identified as a recurrent polymorphism, whereas G335D and T377M were the predominant RT connection substitutions. Similarly, Ngcapu et al. (2017) identified several RNase H substitutions among treatment-experienced individuals with HIV-1C, although only E529D remained significantly associated with treatment after correction for multiple comparisons [28]. Compared with RT connection polymorphisms, recurrent RNase H polymorphisms were less frequently observed in our cohort. These differences may reflect variation in study populations, treatment histories, ART regimens, sampling periods, study objectives and sample size. Furthermore, although several RT connection and RNase H mutations have been described in HIV-1B, and mutations such as A371V and T377M were also observed in the present study, the overall polymorphism profile differed, suggesting that subtype-specific genetic backgrounds and differences in treatment histories may influence the accumulation of extended RT variation [9,10,13,15,30]. Collectively, these findings highlight the need for continued investigation of the extended RT region in HIV-1C to better understand subtype-specific patterns of variation and their potential clinical significance.

Co-occurrence analysis demonstrated that RT connection polymorphisms were not randomly distributed. Instead, mutations clustered into reproducible combinations centred on G335D and T377M, with hierarchical clustering revealing distinct mutational modules. These findings are consistent with previous studies suggesting that RT connection mutations may evolve in coordinated patterns capable of modifying RT function, polymerase processivity and RNase H activity [13,15]. Although these clustering patterns should be interpreted cautiously given the relatively small sample size, they suggest that these substitutions may not occur independently. However, the descriptive cross-sectional design precludes conclusions regarding whether these patterns reflect coordinated viral adaptation or causal biological interactions and therefore require confirmation in larger studies.

The identification of accessory mutation clusters previously associated with reduced RT inhibitor susceptibility is consistent with previous reports and generates hypotheses for further investigation. T377M–T470A, A371V–E399D, T377M–E399D and T377M–A360T were detected in sequences of PWH experiencing VF. These combinations have previously been associated with reduced RT inhibitor susceptibility; however, the present study cannot determine whether they influence drug susceptibility in HIV-1C. Several of these clusters were identified in sequences lacking classical RT DRMs, suggesting that accessory mutations may provide alternative evolutionary pathways supporting continued viral replication under antiretroviral drug pressure [6,8], although their direct contribution to drug resistance cannot be determined from the present study. These findings, however, highlight the genetic complexity of the extended RT region in HIV-1C.

Most participants had extensive treatment histories involving sequential RTIs, PIs and, more recently, dolutegravir-containing regimens. PR and IN resistance mutations remained relatively uncommon compared with the extensive variation observed within the RT connection domain. This observation suggests that long-term selective pressure may continue to shape the genetic variation within the extended RT region despite changes in treatment regimens [10]. However, the present study was not designed to evaluate associations between specific RT connection or RNase H polymorphisms and cumulative exposure to individual antiretroviral drug classes. Such analyses, together with detailed treatment duration and adherence data, would provide valuable insight into the evolutionary drivers of these polymorphisms and should be explored in future studies.

This is one of the first studies in Botswana to comprehensively characterise RT connection and RNase H polymorphisms using ONT sequencing while integrating historical Sanger resistance data, minority variants and accessory mutation clusters. Collectively, these findings add to the growing body of evidence that viral evolution in HIV-1C extends beyond the classical RT domain and suggest that variation within the RT connection and RNase H domains may represent an underexplored component of the genetic structure associated with VF. Although the relatively small sample size, the absence of a comparator group of virally suppressed individuals and the lack of detailed adherence data limit the interpretation and generalisability of these findings, subtype-specific background frequency data from the Stanford HIV Drug Resistance Database provided additional context for interpreting the observed polymorphism profiles. In addition, summary ONT sequencing quality metrics, including mean read depth, Q-scores and homopolymer error rates, were not retained by the analysis pipeline and could therefore not be reported. However, variant calling was performed using predefined quality thresholds implemented within the HIVgenomeR™ v2.0 pipeline to support the reliability of the reported variants. These findings should therefore be interpreted as preliminary and warrant confirmation in larger studies. Functional investigations and larger prospective cohorts will be required to determine whether the observed polymorphism patterns influence RT inhibitor susceptibility or contribute to virologic failure.

## 5. Conclusions

ONT sequencing improved the detection of classical RT DRMs and minority variants in TE PWH experiencing VF. RT connection and RNase H polymorphisms were prevalent across the cohort, including in sequences without detectable RT DRMs. Recurrent polymorphisms and accessory mutation clusters were observed, suggesting that variation within the extended RT domains may occur in non-random patterns. However, given the descriptive cross-sectional design and limited sample size, these observations should be considered preliminary and require confirmation in larger, well-characterised cohorts.

The predominance of HIV-1C consensus residues such as G335D further highlights the importance of subtype-specific interpretation when assessing RT variation in resistance studies. Overall, this study provides pilot data supporting further investigation of the RT connection and RNase H domains as underexplored regions of HIV-1C genetic variation that might explain some HIV drug resistance. Larger studies incorporating functional analyses will be required to determine whether the observed polymorphism patterns influence RT inhibitor susceptibility or contribute to VF.

## Figures and Tables

**Table 1:** Comprehensive resistance profiles of the study cohort.

Seq ID	Historical Sanger Classification	Final ONT Classification	ART Regimen (Most Recent)	Protease Inhibitors (PIs)	Integrase Inhibitors (INSTs)	RT (RT Pol)		RT Connection (Polymorphisms)	RNase H (Polymorphisms)
						Major DRMs	Minor Variants (>4.5% to <20%)		
1	DRM−	DRM− (minority variant)	ABC/3TC/DTG	T74S, K20R	–	–	K103A (4.6%)	A376S	–
2	DRM+	DRM+	TDF/3TC/DTG/DRV/r	T74S	–	G190A, S68G, K101E, T215L	V75I (8.9%), T69A (6.8%)	G335D	–
3	DRM−	DRM+	TDF/3TC/DTG	–	G140A (5.0%)	M41I	–	T377M, T400I	–
4	DRM−	DRM− (no DRM)	EFV/FTC/TDF	K20R	–	–	–	A376S, T377M, G335D	–
5	DRM−	DRM− (minority variant)	TLD	–	V151A (5.1%)	–	V75I (7.4%), G190V (6.5%)	T377M, G335D	T470A
6	DRM+	DRM+	TLD	–	G140A (5.3%)	E138K	T69A (5.6%)	E399D, G335D, A371V, T400L, T377L	–
7	DRM+	DRM+	TLD + DTG + DRV/r (salvage therapy)	T74S, K20R, V821 (18.5%)	V151A (9.7%)	V179D, Y181C, K103R, K219E, T215F, E138Q	V108I (18.9%), T215S (6.2%), K101E (5.7%)	T377M, G335D, E399D	–
8	DRM+	DRM+	3TC/ABC/DTG	–	S230N, Y143H (4.8%)	V90I, E138A, G190A, K70T, K101E	–	G335D	–
9	DRM−	DRM+	3TC/TDF/DTG	T74S	–	V179I	–	G335D, A360T, T377M	–
10	DRM−	DRM− (no DRM)	TLD	T74S, K20M	G140A (7.5%)	–	–	T377M, G335D	–
11	DRM+	DRM+	TDF/FTC/DTG	–	–	K103R, K238R	V106I (6.8%), K238T (5.4%)	A376S, A371V, G335D	–
12	DRM−	DRM+	TDF/FTC/DRV/r/DTG	L10I, K20M	–	T69A, T69N	–	G335D, A376S, T400I, T377M	K558R
13	DRM−	DRM− (minority variant)	TDF/FTC/DTG	–	–	–	Y115F (9.4%)	T377M, G335D	K558R (24.0%) *
14	DRM−	DRM+	EFV/FTC/TDF	–	–	E138A, V179D	K101E (7.5%)	G335D	–
15	DRM−	DRM− (minority variant)	TLD	–	–	–	T69A (5.2%)	G335D, E339D, T400I	–
16	DRM−	DRM+	TLD	–	–	K101A	T69A (5.0%)	T377M, T400L	–

ABC, abacavir; 3TC, lamivudine; TDF, tenofovir disoproxil fumarate; DTG, dolutegravir; DRV/r, darunavir/ritonavir; EFV, efavirenz; FTC, emtricitabine; TLD, TDF + 3TC + DTG; –, none detected. * Minority variant (>4.5% to <20%), Consensus-level variant (≥20%).

## Data Availability

The data presented in this study are not publicly available due to ethical restrictions. De-identified data may be available from the corresponding author upon reasonable request and with approval from the relevant institutional review boards.

## Supplementary Materials

The following supporting information can be downloaded at: https://www.mdpi.com/article/10.3390/biomedicines14091915/s1, Table S1: Complete antiretroviral treatment histories.

## Abbreviations

The following abbreviations are used in this manuscript:

ART	Antiretroviral therapy
VF	Virologic failure
HIV	Human immunodeficiency virus
SSA	Sub-Saharan Africa
HIV-1C	Human immunodeficiency virus type 1 subtype C
PR	Protease
IN	Integrase
RT	Reverse transcriptase
DRMs	Drug resistance mutations
PIs	Protease inhibitors
INSTIs	Integrase strand transfer inhibitors
NRTIs	Nucleoside reverse transcriptase inhibitors
NNRTIs	Non-nucleoside reverse transcriptase inhibitors
PWH	People with HIV
NGS	Next generation sequencing
TE	Treatment-experienced
VL	Viral load
DRM+	Drug resistance mutation-positive
DRM−	Lacking classical reverse transcriptase drug resistance mutations
ONT	Oxford nanopore technologies
RTI	Reverse transcriptase inhibitor
