# Supplementary material for: Reverse Transcriptase Connection and RNase H Domain Variation in HIV-1 Subtype C Among Individuals with Virologic Failure in Botswana
Source: Biomedicines. 2026 Aug 26;14(9):1915. doi: 10.3390/biomedicines14091915 (PMC13604211; doi:10.3390/biomedicines14091915)
Supplement: Supplementary file 1 [file biomedicines-14-01915-s001.zip › biomedicines-4458466-supplementary.pdf]

Table S1. Complete antiretroviral treatment histories

Sequence ID	Regimen history (chronological order)
**1**	ABC/3TC/DTG
**2**	1st line UNK, 2024-CURRENT TDF/3TC/DTG/DAR/r
**3**	2004 AZT/3TC EPV, 2017-CURRENT TDF/3TC/DTG
**4**	1st line ABC+3TC+DTG, 2nd line TDF+FTC/NVP, 3rd line EFV/FTC/TDF
**5**	2013 AZT/3TC/EFV, 2016 TDF+FTC +LPV/r, 2017 TDF+FTC/DTG, 2020 TLD
**6**	2004 AZT+3TC/NVP, 2011 TDF/FTC/LPV/r, 2017 AZT+3TC+DRV/r/DTG, March 2018 TDF/FTC/DTG, May 2018 ATV/r+DTG, CURRENT TLD
**7**	TLD+DTG+DRV/r (salvage therapy)
**8**	2004-2011 AZT/3TC/NVP, 2011-2017 3TC/TDF/LPV/r, 2017-2018 3TC/TDF/DTG, 2018-CURRENT 3TC/ABC/DTG
**9**	2018-CURRENT 3TC/TDF/DTG
**10**	TLD
**11**	Unsuppressed x 3 on TDF/FTC/DTG
**12**	2001 MTCT, 2001 d4T/ddI/?, 2002 AZT/3TC/EFV, 2004 3TC/SQV/r, 2009 TDF/FTC/DRV/r/RAL, 2015 TDF/FTC/DRV/r, 2017 TDF/FTC/DRV/r/DTG
**13**	CURRENT TDF/FTC/DTG
**14**	1st line ABC+3TC+DTG, 2nd line TDF+FTC/NVP, 3rd line EFV/FTC/TDF
**15**	2008-2010 AZT/3TC/NVP, 2010-2016 TDF/FTC/LPV/r, 2016-2017 TDF/FTC/ATZ/r, 2017-2019 TDF/FTC/DTG, 2019-CURRENT TLD
**16**	TLD

ABC, abacavir; 3TC, lamivudine; TDF, tenofovir disoproxil fumarate; DTG, dolutegravir; DAR/r, darunavir/ritonavir; EFV, efavirenz; FTC, emtricitabine; AZT, zidovudine; d4T, stavudine; ddI, didanosine; NVP, nevirapine; LPV/r, lopinavir/ritonavir; ATV/r, atazanavir/ritonavir; SQV/r, saquinavir/ritonavir; RAL, raltegravir; TLD, tenofovir disoproxil fumarate / lamivudine / dolutegravir regimen
